# Association of Inner Retinal Thickness with Prevalent Dementia and Brain Atrophy in a General Older Population: The Hisayama Study

**DOI:** 10.1016/j.xops.2022.100157

**Published:** 2022-04-19

**Authors:** Emi Ueda, Naoki Hirabayashi, Tomoyuki Ohara, Jun Hata, Takanori Honda, Kohta Fujiwara, Yoshihiko Furuta, Mao Shibata, Sawako Hashimoto, Shun Nakamura, Taro Nakazawa, Tomohiro Nakao, Takanari Kitazono, Toshiharu Ninomiya, Koh-Hei Sonoda

**Affiliations:** 1 Department of Epidemiology and Public Health, Graduate School of Medical Sciences, Kyushu University, Fukuoka, Japan; 2 Department of Ophthalmology, Graduate School of Medical Sciences, Kyushu University, Fukuoka, Japan; 3 Ito Clinic, Kyushu University, Fukuoka, Japan; 4 Department of Psychosomatic Medicine, Graduate School of Medical Sciences, Kyushu University, Fukuoka, Japan; 5 Department of Neuropsychiatry, Graduate School of Medical Sciences, Kyushu University, Fukuoka, Japan; 6 Center for Cohort Studies, Graduate School of Medical Sciences, Kyushu University, Fukuoka, Japan; 7 Department of Medicine and Clinical Science, Graduate School of Medical Sciences, Kyushu University, Fukuoka, Japan

**Keywords:** Alzheimer’s disease, Brain atrophy, Dementia, Ganglion cell-inner plexiform layer, Population-based study, Retinal nerve fiber layer, AD, Alzheimer’s disease, CI, confidence interval, FDR, false discovery rate, GC-IPL, ganglion cell-inner plexiform layer, ICV, intracranial volume, MMSE, Mini-Mental State Examination, MRI, magnetic resonance imaging, OR, odds ratio, RNFL, retinal nerve fiber layer, SD, standard deviation, SS-OCT, swept-source OCT, VBM, voxel-based morphometry

## Abstract

**Purpose:**

To assess the association of inner retinal thickness with prevalent dementia and regional brain atrophy in a general older population of Japanese.

**Design:**

Population-based, cross-sectional study.

**Participants:**

A total of 1078 residents aged 65 years or older who participated in an eye examination, a comprehensive survey of dementia, and brain magnetic resonance imaging scanning in 2017.

**Methods:**

The thicknesses of the inner retinal layers, namely, the ganglion cell-inner plexiform layer (GC-IPL) and retinal nerve fiber layer (RNFL)—were measured by swept-source OCT (SS-OCT). The association of these retinal thicknesses with the risk of the presence of dementia was estimated using restricted cubic splines and logistic regression models. Regional brain volumes were estimated separately by applying 2 different methods: voxel-based morphometry (VBM) and analysis by FreeSurfer software. The associations of GC-IPL and RNFL thickness with each brain regional volume were analyzed using multiple regression analysis.

**Main Outcome Measure:**

Prevalent dementia and regional brain atrophy.

**Results:**

Among the study participants, 61 participants (5.7%) were diagnosed with dementia. The likelihood of the presence of dementia significantly increased with lower GC-IPL thickness after adjusting for potential confounders (odds ratio, 1.62 [95% confidence interval, 1.30–2.01] per 1 standard deviation decrement in the GC-IPL thickness), but no significant association was observed with RNFL thickness. In the VBM analyses with the multivariable adjustment, lower GC-IPL thickness was significantly associated with lower volume of known brain regions related to cognitive functions (i.e., the hippocampus, amygdala, entorhinal area, and parahippocampal gyrus) and visual functions (i.e., the cuneus, lingual gyrus, and thalamus). Meanwhile, the volume of the thalamus significantly decreased with lower RNFL thickness, but none of the brain regions related to cognitive function exhibited a volume change in association with RNFL thickness. The sensitivity analysis using FreeSurfer analysis also showed that lower GC-IPL thickness was significantly associated with lower regional brain volume/intracranial volume of the hippocampus, amygdala, cuneus, lingual gyrus, and thalamus.

**Conclusions:**

Our findings suggest that the measurement of GC-IPL thickness by SS-OCT, which is a noninvasive, convenient, and reproducible method, might be useful for identifying high-risk individuals with dementia.

Dementia is one of the major causes of disability in older populations, and its medical and economic burdens have been increasing in societies worldwide.^1^ Brain atrophy is a morphological feature of dementia that is known to occur in the preclinical stage of disease,^2^ and magnetic resonance imaging (MRI) studies have reported that patients with dementia or mild cognitive impairment have specific brain atrophy in several regions, including the hippocampus. These data suggest that assessment of regional brain atrophy by MRI may be an image marker for dementia, but brain MRI examination is not always available in some clinical settings, such as at general practices or community health checkups. Therefore, there is need for an alternative imaging measure that is noninvasive, convenient, and inexpensive to assess individuals at high risk for dementia.

Inner retinal layers—namely, the ganglion cell-inner plexiform layer (GC-IPL) and retinal nerve fiber layer (RNFL)—share structural and pathogenic pathways with the brain. Currently, swept-source OCT (SS-OCT) can be used to measure segments of the retinal sublayer, including the GC-IPL and RNFL, noninvasively, quantitatively, and reproducibly.^3^^,^^4^ Several neuropathological studies of individuals with Alzheimer’s disease (AD) and AD model mice have revealed the accumulation of amyloid-β or tau protein in these inner retinal layers.^5^, ^6^, ^7^, ^8^, ^9^, ^10^, ^11^ Clinical and population-based studies^12^^,^^13^ have reported that the thinning of these inner retinal layers is associated with a higher likelihood of prevalent dementia or AD.^14^, ^15^, ^16^, ^17^, ^18^ However, no population-based, cross-sectional study has examined the association between inner retinal thickness and subtypes of dementia. In addition, population-based studies using brain MRI data have reported a close positive association between the thickness of the inner retinal layer and atrophy in specific brain regions related to cognitive function, such as the hippocampus.^19^, ^20^, ^21^ Since recent advanced techniques for imaging analysis, such as voxel-based morphometry (VBM)^22^ and FreeSurfer analysis,^23^ have allowed us to quantitatively measure the brain volume of each anatomic region based on brain MRI data, it is now possible to assess the association between retinal thickness and brain atrophy in multiple regions by using VBM or FreeSurfer. However, to our knowledge only 1 population-based study has investigated the association of GC-IPL and RNFL thickness with the regional brain volumes or the brain atrophy pattern by using quantitative imaging techniques.^24^

The objectives of this study were to elucidate the association of the inner retinal thickness evaluated by using SS-OCT with the risk of the presence of dementia and to identify the brain regions associated with inner retinal layer thickness by applying VBM and FreeSurfer software to brain MRI data from a general older Japanese population.

## Methods

### Study Population

The Hisayama Study is an ongoing population-based prospective longitudinal study of cerebro-cardiovascular diseases that began in 1961 in the town of Hisayama, a suburban community adjacent to the city of Fukuoka in southern Japan.^25^ Since 1985, we have also conducted comprehensive surveys of dementia every 5 to 7 years (i.e., 1985, 1992, 1998, 2005, 2012, and 2017) in older residents of this town to identify the individuals with dementia or disability.^26^^,^^27^ In addition, a survey of eye diseases among residents of this town has been under way since 1998.^28^

In 2017–2018, among 2340 Hisayama residents aged ≥ 65 years, a total of 2202 residents (participation rate = 94.1%) participated in an examination for a screening survey of cognitive function and health status, including a screening for eye disease.^26^ Of these, 1577 participants (71.6%) underwent brain MRI scanning for the present study. Among these 1577 participants, we excluded 7 individuals who did not provide their consent for study participation, 119 individuals whose brain MRI data were considered not applicable for the VBM or FreeSurfer analysis based on visual assessment by the study team (7 with artifacts, 17 with skull stripping errors, 9 with trace errors, 78 with brain infarction, 1 with brain hemorrhage, 1 with chronic subdural hematoma, 1 with brain tumor, 5 with any errors in the evaluation process of the regional brain volumes using the FreeSurfer software for the imaging analysis), 167 individuals without OCT scans, 73 individuals with poor-quality OCT scans, and 133 individuals with eye diseases (age-related macular degeneration, glaucoma, diabetic retinopathy, macular hole, epiretinal membrane, and retinitis pigmentosa). The remaining 1078 participants (447 men and 631 women) were enrolled in the present study.

### Measurements of Inner Retinal Layer Thickness and Other Ophthalmic Examinations

**Figure 1:**
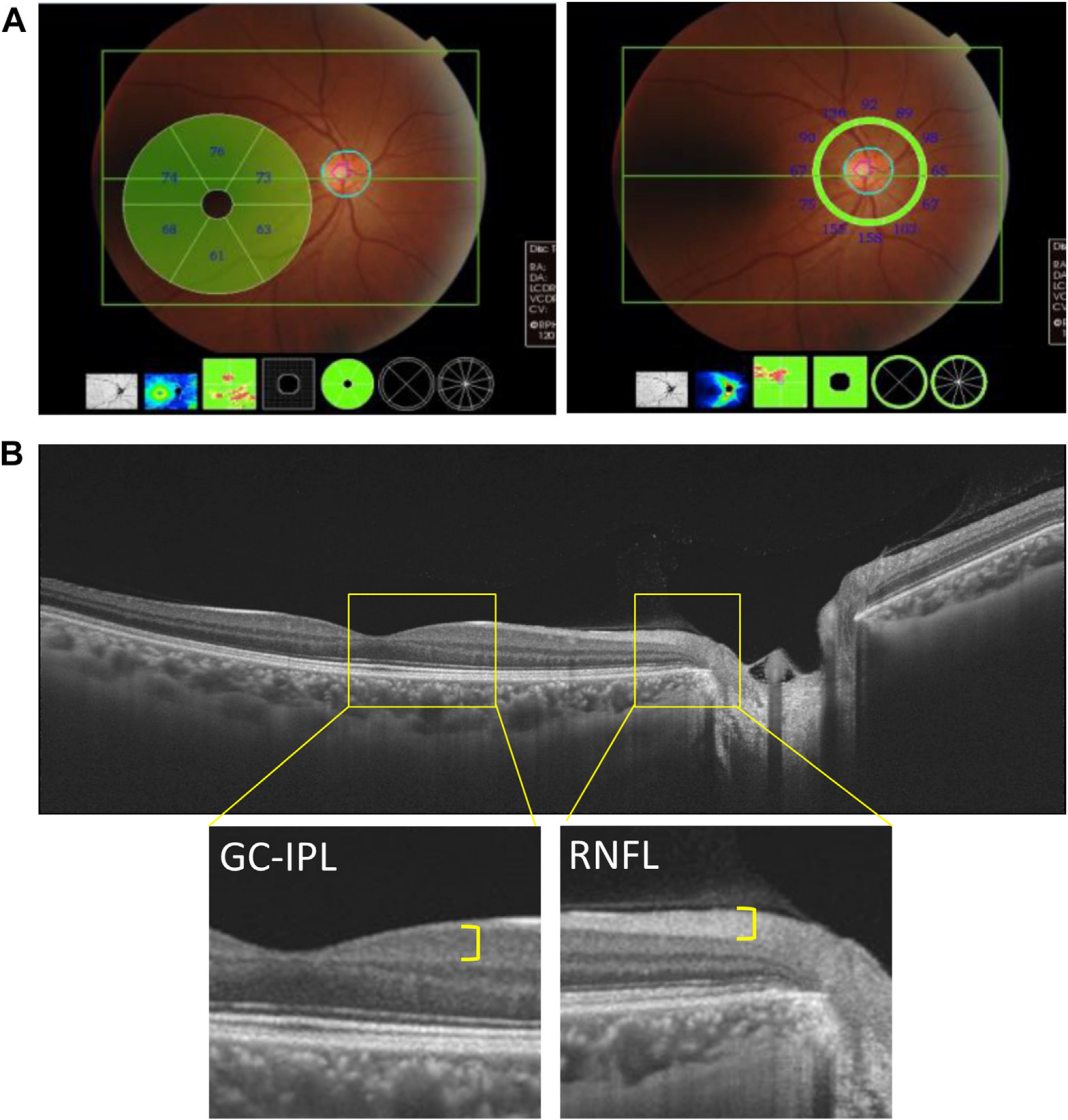
Measurements of ganglion cell–inner plexiform layer (GC-IPL) and retinal nerve fiber layer (RNFL) thickness on swept-source OCT, 2017. **A,** The thickness of the GC-IPL was evaluated with a diameter of 6 mm centered on the fovea, and the thickness of the RNFL was evaluated in a circle with a diameter of 3.4 mm centered on the optic disc. The average of each retinal thickness was calculated and used for the analysis. **B,** Wide scanning over a 12 × 9 mm area was performed at a scan density of 512 A-scans (horizontal) × 128.

Each participant underwent ophthalmic examination after pupil dilatation with 0.5% tropicamide and 0.5% phenylephrine hydrochloride. Imaging was performed using an SS-OCT instrument, the DRI-OCT Triton (Topcon). The principles of OCT have been well described.^29^^,^^30^ We photographed 1 field, centered at a point midway between the temporal edge of the optic disc and the fovea in both eyes. Wide scanning over a 12 × 9-mm area was performed at a scan density of 512 A-scans (horizontal) × 128. The GC-IPL thickness was evaluated in 6 sector grids with a diameter of 6 mm centered on the fovea, and the RNFL thickness was evaluated in a circle with a diameter of 3.4 mm centered on the optic disc, which was divided into 4 sectors (Fig 1). The average thickness was calculated and used for the analysis. Image quality values <30 (as recommended by the manufacturer) were excluded from the analyses. All scans were reviewed, and 2 ophthalmologists (E.U. and K.F.), who were masked to the excluded poor-quality scans, manually corrected the centering of images if necessary. Glaucoma was defined on the basis of the criteria of the International Society for Geographical and Epidemiological Ophthalmology.^31^ Axial length measurements were performed with noncontact partial coherence laser interferometry (OA-2000; Tomey GmbH). We used the information on the right eye for the present analysis.

### Diagnosis of Dementia

In the screening survey of cognitive impairment, we used the Mini-Mental State Examination (MMSE). Individuals who met ≥ 1 of the following criteria underwent a second screening survey for potential cognitive impairment: (1) MMSE score ≤26 points; (2) a score of ≤4 of a total possible 6 points on the delayed recall test components of the MMSE based on the Modified MMSE^32^ to detect suspected cases of cognitive impairment more sensitively (i.e., 3 questions, each scored 2 points if answered correctly without a hint; 1 point if answered correctly with a hint; and 0 points if answered incorrectly with/without a hint); (3) a failed intersecting pentagon-copying test in the MMSE or cube-copying test;^33^ and (4) suspected cases based on the manner of speaking and behavior. For individuals with suspected dementia, secondary comprehensive investigations including Wechsler Memory Scale of logical memory were conducted by expert psychiatrists as previously described.^26^^,^^27^ Dementia was ascertained using the criteria of the Diagnostic and Statistical Manual of Mental Disorders, Revised Third Edition.^34^ Expert psychiatrists and physicians of stroke in the study team adjudicated every case of dementia. We used the criteria of the National Institute of Neurological and Communicative Disorders and Stroke and the Alzheimer’s Disease and Related Disorders Association^35^ and the criteria of the National Institute of Neurological Disorders and Stroke–Association International pour la Recherche et l’Enseignement en Neurosciences^36^ to diagnose AD and vascular dementia, respectively. The clinical information and morphologic examination from neuroimaging were used to define probable or possible dementia subtypes.

### MRI Analysis

#### Image Acquisition

Using a 1.5-Tesla MRI scanner (Intera Pulsar; Philips Medical Systems) with a multichannel head coil, we examined 3-dimensional T1-weighted images, conventional T1- and T2-weighted images, fluid-attenuated inversion recovery, T2∗-weighted images, and magnetic resonance angiographic images of the brain. T1-weighted 3-dimensional images were acquired in the sagittal plane with the following parameters: repetition time 8.5 ms, echo time 4.0 ms, inversion time 1000 ms, flip angle 8°, field of view 240 mm, acquisition matrix 192 × 192, slice thickness 1.2 mm, number of excitations 1.

### Voxel-based Morphometry Preprocessing

T1-weighted 3-dimensional images were segmented into gray matter, white matter, and cerebrospinal fluid by using VBM8 Toolbox version 435 (University of Jena, Germany) in SPM8 (University College London, UK) running in MATLAB (The Mathworks, Inc.). The International Consortium for Brain Mapping template for East Asian brains was used for anatomic settings. We also created the binarized white matter hyperintensity masks for each individual, as previously described.^37^ Because white matter hyperintensities on fluid-attenuated inversion recovery images are often misclassified as gray matter on T1 scans, the gray matter and white matter maps were corrected using the binarized white matter hyperintensity masks. Segmented gray matter images were normalized and modulated to compensate for the volumetric effects of expansion/shrinking in spatial normalization. Last, the images were smoothed with an 8-mm full-width, half-maximum isotropic Gaussian kernel. The intracranial volume (ICV) was calculated as the sum of the gray matter, white matter, and cerebrospinal fluid volumes.

### FreeSurfer Preprocessing

The segmentation and volume measurements of cortical and subcortical brain structures were performed automatically using FreeSurfer software, version 6.0.0 (Harvard University). All the results of the automated segmentation by FreeSurfer were visually checked for accuracy by the study team. We obtained cortical volumes (entorhinal cortex, parahippocampal gyrus, cuneus, and lingual gyrus), subcortical volumes (hippocampus, amygdala, and thalamus), the white matter hyperintensities volume, and the ICV. To correct for head size, the regional brain volume/ICV and white matter hyperintensities volume/ICV ratios were calculated as a percentage of ICV as follows: ([left + right] regional brain volume/ICV) x 100 (%) and the volume of white matter hyperintensities/ICV x 100 (%).

### Risk Factor Measurements

Each subject completed a self-administered questionnaire that included items on educational status, smoking habits, alcohol intake, physical activity, and use of antihypertensive agents, glucose-lowering agents, and lipid-modifying agents. A face-to-face interview was conducted by trained registered nurses for all participants, including those who had difficulty completing or were unable to complete the questionnaire. We defined low education as ≤ 9 formal educational years. Blood pressure was measured 3 times after > 5 minutes of rest in the sitting position, and the mean value of the 3 measurements was calculated. We measured plasma glucose levels by the hexokinase method and defined diabetes as a fasting plasma glucose level ≥ 7.0 mmol/l, casual or 2-hour postload plasma glucose level by 75 g oral glucose tolerance test ≥ 11.1 mmol/l, or use of glucose-lowering agents. We also measured serum total cholesterol levels enzymatically and defined hypercholesterolemia as serum cholesterol ≥ 5.69 mmol/l or use of lipid-modifying agents. We measured body height and weight in light clothing without shoes and calculated the body mass index (kg/m^2^). We classified alcohol intake and smoking habits as current habitual use or not. Regular exercise was defined as engaged in sports or other forms of exercise at least 3 times per week during leisure time.

### Statistical Methods

We calculated the Spearman correlation coefficients between GC-IPL or RNFL thickness and clinical factors of the study population. To show the shape of the associations between each inner retinal layer thickness and the risk of the presence of dementia, we used a logistic regression analysis with restricted cubic splines.^38^ In this analysis, 3 knots were placed at the 10th, 50th, and 90th percentiles of the retinal thickness levels (for GC-IPL: 59.6, 68.2, and 74.8 μm; for RNFL: 78.6, 100.4, and 114.8 μm), and the 50th percentile was set as the reference value. We also tested the nonlinear association between the thickness of each inner retinal layer and the presence of dementia based on the likelihood ratio test, comparing the log-likelihood of the model with a linear term to the log-likelihood of a model with cubic spline terms. The odds ratios (ORs) with their 95% confidence intervals (CIs) of the presence of dementia per every 1 standard deviation (SD) decrement in the thickness of each inner retinal layer were estimated by using a logistic regression analysis. In the multivariable-adjusted analysis, the following covariates were included in the relevant model: age, sex, education status, systolic blood pressure, use of antihypertensive drugs, diabetes, serum total cholesterol, body mass index, cerebrovascular lesions on MRI, smoking habits, drinking habits, regular exercise, and axial length. In the analysis with VBM, the association of each retinal thickness with regional brain volume was analyzed using a multiple regression analysis covarying for the mentioned confounding factors and the ICV. We applied explicit masking using a gray matter template included in the VBM8 Toolbox (Template_6_IXI550_MNI152.nii) with gray matter values of > 0.05 and voxel wise absolute masking with a threshold of 0.01. To perform multiple comparisons correction, all statistical maps were thresholded at *P* < 0.001 for uncorrected at the voxel level and *P* < 0.05 for familywise error correction at the cluster level.^39^ The color intensity represents T-statistic values, and there is a strong positive correlation with the intensity of the yellow color. In addition, an analysis with FreeSurfer automated segmentation was performed to assess the association of each inner retinal thickness with the volumes of the known brain regions related to cognitive function (the hippocampus, amygdala, entorhinal cortex, and parahippocampal gyrus)^40^^,^^41^ and visual function (the cuneus, lingual gyrus, and thalamus)^42^^,^^43^ among retinal thickness-related brain regions detected by VBM analysis. The white matter hyperintensities volume was natural log transformed because its distribution was skewed. Regression coefficients (β) with 95% CIs per every 1 SD decrement in the thickness of each inner retinal layer with the regional brain volumes/ICV ratio or white matter hyperintensities volume/ICV ratio were computed by using a multiple linear regression analysis with the above-mentioned confounding factors. False discovery rate (FDR) correction^44^ was performed to verify the multiple comparisons for which a significance level with a q value of FDR correction was defined as < 0.10.^45^ SAS software (v.9.4; SAS Institute, Inc.) was used to perform all statistical analyses. A 2-tailed value of *P* < 0.05 was considered statistically significant in all analyses.

### Ethical Considerations

This study was approved by the Kyushu University Institutional Review Board for Clinical Research and was carried out in accordance with the Declaration of Helsinki. Informed consent was obtained from all participants.

## Results

The clinical characteristics of the study population and Spearman's correlation coefficients between each retinal layer thickness and risk factor are shown in Table 1. The mean (SD) values of GC-IPL thickness and RNFL thickness were 67.3 (7.4) μm and 98.5 (17.9) μm, respectively. With regard to the correlation between retinal layer thickness and each risk factor, GC-IPL and RNFL thickness were negatively correlated with age, systolic blood pressure, use of antihypertensive agents, the presence of diabetes mellitus, and axial length, but positively correlated with serum total cholesterol. There was a negative correlation between GC-IPL thickness and the presence of cerebrovascular lesions on MRI.

**Table 1:** Clinical Characteristics of the Total Study Population and the Spearman Correlation Coefficients of the GC-IPL and RNFL Thickness with Each Clinical Factor, 2017

Variables	Total Population (n = 1078)	GC-IPL Thickness	RNFL Thickness
Age, yrs	74 (6.5)	−0.17∗	−0.12∗
Men, %	41.5	0.01	0.10∗
Education ≤9 yrs, %	28.0	−0.05	−0.04
Systolic blood pressure, mmHg	129.5 (17.6)	−0.06∗	−0.09∗
Use of antihypertensive agents, %	52.0	−0.11∗	−0.09∗
Diabetes mellitus, %	22.5	−0.09∗	−0.06∗
Serum total cholesterol, mmol/l	5.36 (0.95)	0.10∗	0.11∗
Body mass index, kg/m^2^	23.3 (3.5)	−0.02	0.003
Cerebrovascular lesions on MRI, %	28.4	−0.06∗	−0.06
Smoking habits, %	40.4	0.006	−0.01
Drinking habits, %	60.8	−0.01	−0.04
Regular exercise, %	22.4	−0.01	0.004
Axial length, mm	23.6 (1.3)	−0.22∗	−0.28∗

GC-IPL = ganglion cell-inner plexiform layer; MRI = magnetic resonance imaging; RNFL = retinal nerve fiber layer.

All values for the total population are given as means (standard deviations) or as frequencies.

Cerebrovascular lesions were defined as brain infarction or hemorrhage on MRI regardless of the presence of absence of neurological symptoms.

∗ *P* < 0.05.

Among the study participants, 61 (5.7%) were diagnosed as having dementia. Regarding the subtypes of dementia, 52 participants were diagnosed with AD, 3 participants were diagnosed with a mixed type of AD and vascular dementia, 1 participant was diagnosed with a mixed type of dementia with AD and dementia with Lewy bodies, 2 participants were diagnosed with vascular dementia, 1 participant was diagnosed with dementia with Lewy bodies, 1 participant was diagnosed with hypoxic ischemic encephalopathy, and 1 participant was diagnosed with frontotemporal lobar degeneration (Supplemental Table S1). In the analysis for AD, participants with mixed types of dementia with AD were counted as having AD.

**Figure 2:**
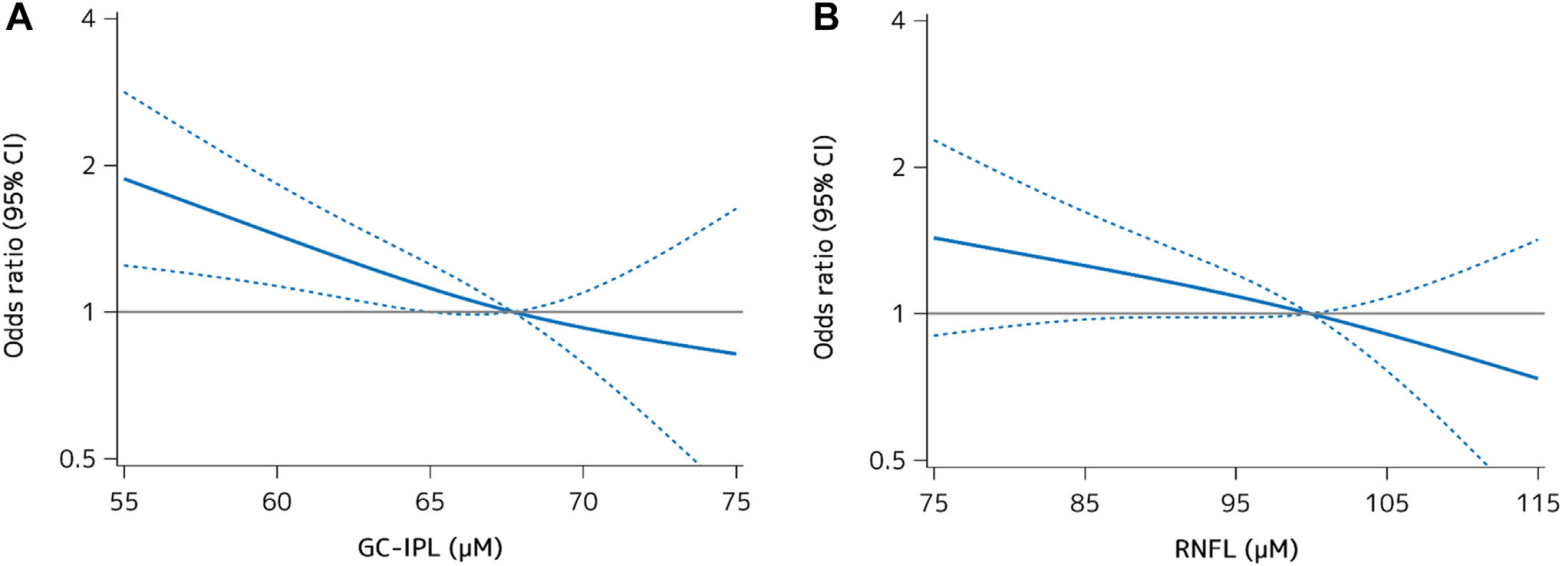
Logistic regression analysis with restricted cubic splines for the association between each retinal layer and the likelihood of dementia, 2017. The odds ratios (ORs) for prevalent dementia tended to increase linearly with the decrease of ganglion cell–inner plexiform layer (GC-IPL) thickness **(A)** and retinal nerve fiber layer (RNFL) thickness **(B)**. **Solid lines** represent the OR, and **dashed lines** represent the 95% confidence interval (CI). Knots were placed at the 10th, 50th, and 90th percentiles (for GC-IPL: 59.6, 68.2, and 74.8 μm; for RNFL: 78.6, 100.4, and 114.8 μm). A reference point was set at 68.2 μm of GC-IPL and 100.4 μm of RNFL. The ORs were adjusted for age, sex, education, systolic blood pressure, use of antihypertensive agents, diabetes, serum total cholesterol, body mass index, cerebrovascular lesions on magnetic resonance imaging, smoking habits, drinking habits, regular exercise, and axial length.

To assess the shape of the associations between each retinal thickness and prevalent dementia, we performed a logistic regression analysis with restricted cubic spline analyses (Fig 2). As a consequence, both GC-IPL thickness (Fig 2A) and RNFL thickness (Fig 2B) showed an almost linear negative association with the likelihood of the presence of dementia after adjusting for age, sex, education, systolic blood pressure, use of antihypertensive agents, diabetes, serum total cholesterol, body mass index, cerebrovascular lesions on MRI, smoking habits, drinking habits, regular exercise, and axial length (GC-IPL: *P* for nonlinearity = 0.55; RNFL: *P* for nonlinearity = 0.90). Table 2 demonstrates the age- and sex-adjusted and multivariable-adjusted ORs for the presence of all-cause dementia and AD per every 1 SD decrement in GC-IPL and RNFL thickness. The multivariable-adjusted ORs for the presence of all-cause dementia and AD significantly increased with lower GC-IPL thickness after adjusting for potential confounders (per 1 SD decrement: OR, 1.62, 95% CI, 1.30−2.01, *P* < 0.001 for all-cause dementia; OR, 1.54, 95% CI, 1.22−1.94, *P* < 0.001 for AD). On the other hand, no significant associations were observed between RNFL thickness and prevalent dementia in either the age- and sex-adjusted or multivariable-adjusted models. We also examined the association between the inner retinal thickness and the presence of all-cause dementia in the right and left eyes and for each sector related to the fovea and the optic disc. The results showed that overall and sector-specific GC-IPL thickness for the whole eye and for each sector, but not RNFL thickness, were significantly associated with the presence of dementia in both the right and left eyes. There was no material difference in theses associations between the whole right eye and whole left eye, or across the sectors in either the right or left eye (Supplemental Table S2).

**Table 2:** OR for the Presence of all-Cause Dementia and AD and per Every 1 SD Decrement in the GC-IPL and RNFL Thickness, 2017

	No. of Events/Individuals at Risk	Age- and Sex-Adjusted		Multivariable-Adjusted∗	
		OR (95% CI) per Every 1 SD Decrement	P	OR (95% CI) per Every 1 SD Decrement	P
**All-Cause Dementia**					
GC-IPL thickness	61/1078	1.52 (1.25−1.87)	<0.001	1.62 (1.30−2.01)	<0.001
RNFL thickness	61/1078	1.24 (0.97−1.60)	0.10	1.26 (0.96−1.68)	0.10
**Alzheimer’s Disease**					
GC-IPL thickness	56/1078	1.45 (1.17−1.79)	<0.001	1.54 (1.22−1.94)	<0.001
RNFL thickness	56/1078	1.20 (0.93−1.54)	0.17	1.20 (0.90−1.60)	0.21

CI = confidence interval; GC-IPL = ganglion cell-inner plexiform layer; OR = odds ratio; RNFL = retinal nerve fiber layer; SD = standard deviation.

The SDs of GC-IPL thickness and RNFL thickness were 7.4 and 17.9, respectively.

∗ The values were adjusted for age, sex, education, systolic blood pressure, use of antihypertensive agents, diabetes, serum total cholesterol, body mass index, cerebrovascular lesions on magnetic resonance imaging, smoking habits, drinking habits, regular exercise, and axial length.

**Figure 3:**
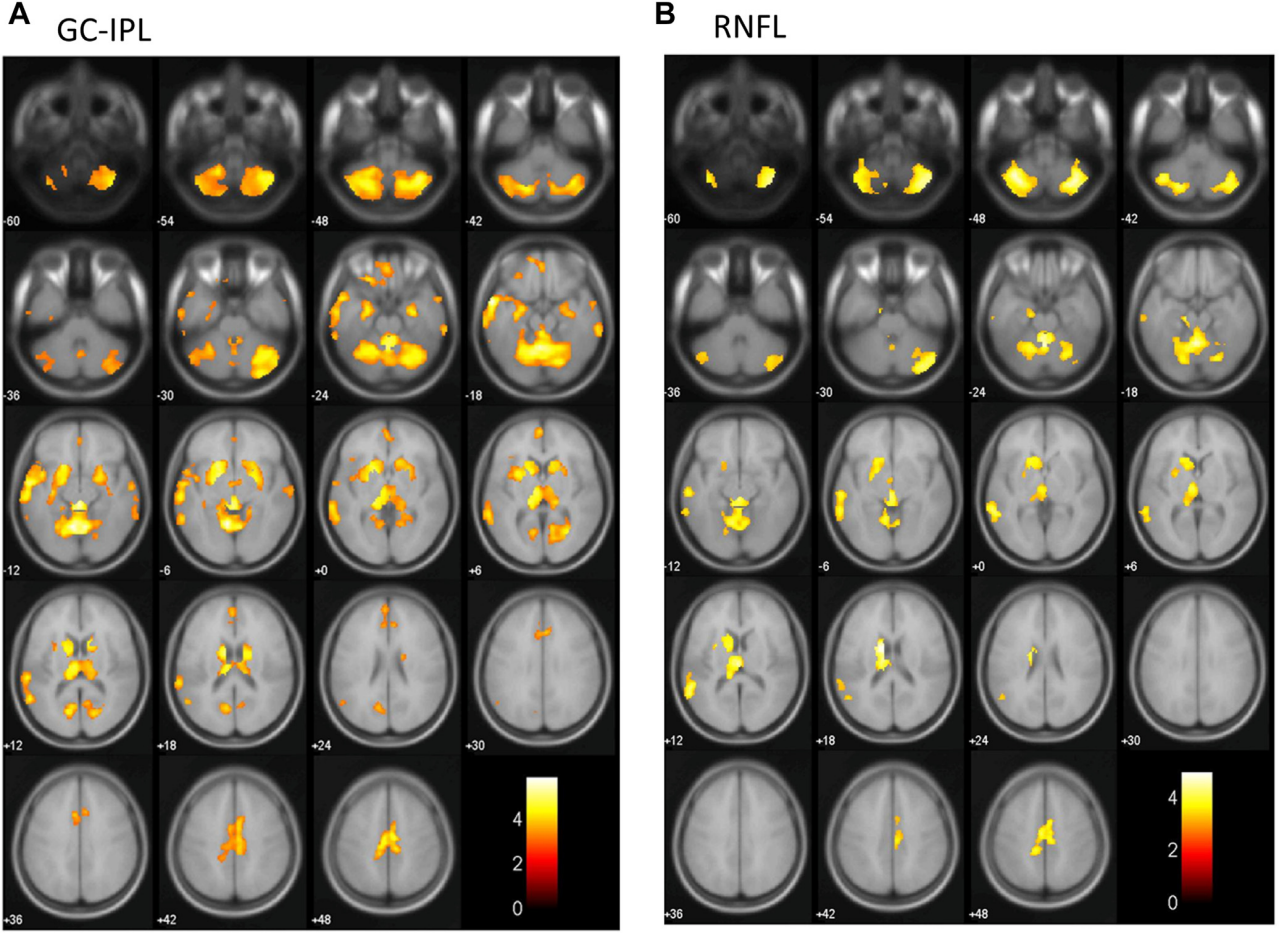
Brain atrophy patterns that were correlated with each retinal layer by voxel-based morphometry (VBM), 2017. The yellow-red heat map shows the brain regions associated with a thinner retinal layer in the total study population. We used a multiple regression analysis with adjustment for age, sex, education, systolic blood pressure, use of antihypertensive agents, diabetes, serum total cholesterol, body mass index, cerebrovascular lesions on magnetic resonance imaging, smoking habits, drinking habits, regular exercise, axial length, and intracranial volume to assess the association of retinal thickness with the regional brain volume estimated by using VBM. The color intensity represents T-statistic values, and there was a strong positive correlation between these values and the intensity of the yellow color. **A,** Ganglion cell-inner plexiform layer (GC-IPL): The region of brain atrophy mainly involved the bilateral hippocampus, bilateral amygdala, left entorhinal area, left parahippocampal gyrus, left cuneus, bilateral lingual gyrus, bilateral thalamus, bilateral calcarine cortex, bilateral middle temporal gyrus, left superior temporal gyrus, bilateral medial frontal lobe, cingulum, bilateral basal ganglia, and cerebellum. **B,** Retinal nerve fiber layer (RNFL): The region of brain atrophy mainly involved the left thalamus, left middle temporal gyrus, left superior temporal gyrus, cingulum, left basal ganglia, and cerebellum.

**Figure 4:**
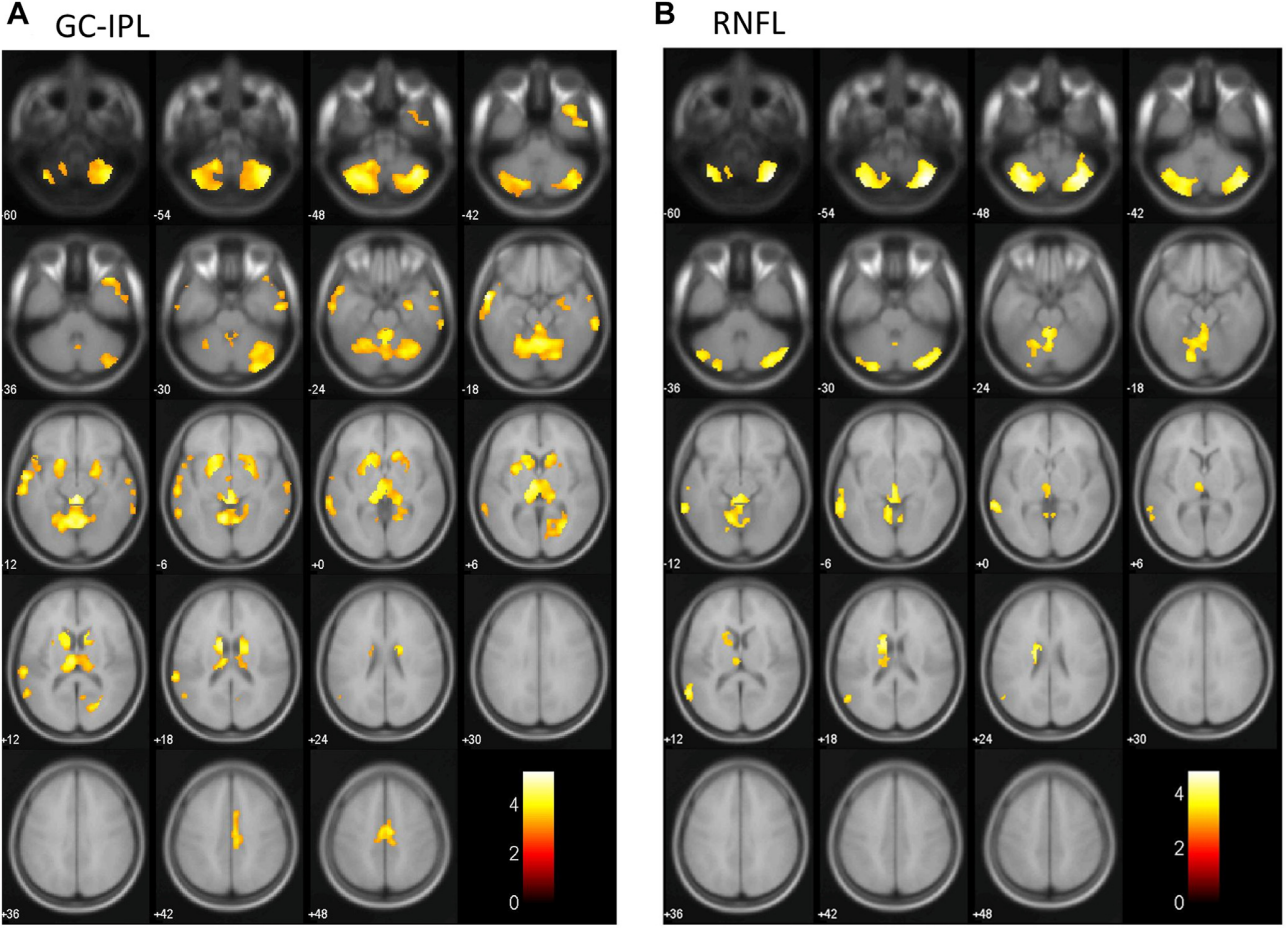
Brain atrophy patterns that were correlated with each retinal layer in individuals without dementia by voxel-based morphometry (VBM), 2017. The yellow-red heat map shows brain regions associated with a thinner retinal layer after excluding individuals with dementia from the total study population. We used a multiple regression analysis with adjustment for age, sex, education, systolic blood pressure, use of antihypertensive agents, diabetes, serum total cholesterol, body mass index, cerebrovascular lesions on magnetic resonance imaging, smoking habits, drinking habits, regular exercise, axial length, and intracranial volume. The color intensity represents T-statistic values, and there was a strong positive correlation between these values and the intensity of the yellow color. **A,** Ganglion cell-inner plexiform layer (GC-IPL): The region of brain atrophy mainly involved the right hippocampus, right amygdala, bilateral lingual gyrus, bilateral thalamus, right calcarine cortex, bilateral middle temporal gyrus, left superior temporal gyrus, bilateral basal ganglia, cingulum, and cerebellum. **B,** Retinal nerve fiber layer (RNFL): The region of brain atrophy mainly involved the left thalamus, right calcarine cortex, left middle temporal gyrus, left basal ganglia, and cerebellum.

We evaluated the association between each inner retinal layer thickness and each brain region volume estimated by using VBM analysis with adjustment for confounding factors (Fig 3). Lower GC-IPL thickness was significantly correlated with a lower volume of the bilateral hippocampus, bilateral amygdala, left entorhinal area, left parahippocampal gyrus, left cuneus, bilateral lingual gyrus, bilateral thalamus, bilateral calcarine cortex, bilateral middle temporal gyrus, left superior temporal gyrus, bilateral medial frontal lobe, cingulum, bilateral basal ganglia, and cerebellum (Fig 3A). Meanwhile, lower RNFL thickness was significantly correlated with a lower volume of the left thalamus, left middle temporal gyrus, left superior temporal gyrus, cingulum, left basal ganglia, and cerebellum (Fig 3B). In the sensitivity analyses after excluding individuals with dementia, similar brain regions were also identified as regions showing lower brain volume with decreasing thickness of GC-IPL (the right hippocampus, right amygdala, bilateral lingual gyrus, bilateral thalamus, right calcarine cortex, bilateral middle temporal gyrus, left superior temporal gyrus, cingulum, bilateral basal ganglia, and cerebellum) (Fig 4A) and RNFL (the left thalamus, left middle temporal gyrus, left basal ganglia, and cerebellum) (Fig 4B).

Furthermore, we investigated the association between each inner retinal thickness and known brain regions related to cognitive function (the hippocampus, amygdala, entorhinal cortex, and parahippocampal gyrus)^37^^,^^38^ and visual function (the cuneus, lingual gyrus, and thalamus)^39^^,^^40^ among retinal thickness-related brain regions detected by VBM analysis, and between each inner retinal thickness and white matter hyperintensities by using FreeSurfer automated segmentation analysis (Table 3). Lower GC-IPL thickness was significantly associated with lower regional brain volumes/ICV ratio of the hippocampus and amygdala (per 1 SD decrement: hippocampus, β = −0.0040, *P* = 0.01, q values of FDR correction = 0.02; amygdala, β = −0.0019, *P* = 0.01, q values = 0.02), but RNFL thickness was not. The regional brain volumes of the cuneus, lingual gyrus, and thalamus decreased with lower GC-IPL thickness (per 1 SD decrement: cuneus, β = −0.0047, *P* = 0.003, q values = 0.03; lingual gyrus, β = −0.0066, *P* = 0.03, q values = 0.045; thalamus, β = −0.0054, *P* = 0.01, q values = 0.03), as well as lower RNFL thickness (per 1 SD decrement: cuneus, β = −0.0051, *P* = 0.002, q values = 0.01; thalamus, β = −0.0052, *P* = 0.01, q values = 0.04). However, no significant associations with the white matter hyperintensities volume/ICV ratio were observed for GC-IPL thickness or RNFL thickness.

**Table 3:** Association of the GC-IPL and RNFL Thickness with the Regional Brain Volume/ICV, Estimated by the FreeSurfer Software, 2017

	GC-IPL Thickness per Every 1 SD Decrement				RNFL Thickness per Every 1 SD Decrement			
	β	95% CI	P Value	q Value of FDR Correction	β	95% CI	P Value	q Value of FDR Correction
Total Study Population (n = 1078)								
Hippocampus	−0.0040	(−0.0072 to −0.0009)	0.01	0.02	−0.0025	(−0.0057 to 0.0006)	0.12	0.31
Amygdala	−0.0019	(−0.0032 to −0.0005)	0.01	0.02	−0.0009	(−0.0023 to 0.0006)	0.24	0.32
Entorhinal cortex	−0.0011	(−0.0039 to 0.0017)	0.46	0.46	0.0001	(−0.0027 to 0.0029)	0.94	0.94
Parahippocampal gyrus	−0.0008	(−0.0029 to 0.0013)	0.45	0.51	0.0007	(−0.0014 to 0.0028)	0.53	0.60
Cuneus	−0.0047	(−0.0078 to −0.0016)	0.003	0.03	−0.0051	(−0.0082 to –0.0020)	0.002	0.01
Lingual gyrus	−0.0066	(−0.0126 to −0.0007)	0.03	0.045	−0.0046	(−0.0106 to 0.0014)	0.14	0.27
Thalamus	−0.0054	(−0.0092 to −0.0015)	0.01	0.03	−0.0052	(−0.0091 to −0.0012)	0.01	0.04
White matter hyperintensities	0.0388	(−0.0091 to 0.0787)	0.10	0.10	0.0269	(−0.0134 to 0.0672)	0.19	0.30

CI = confidence interval; FDR = false discovery rate; GC-IPL = ganglion cell-inner plexiform layer; ICV = intracranial volume; RNFL = retinal nerve fiber layer; SD = standard deviation.

Regional brain volume is calculated as a percentage of ICV as follows: (regional brain volume/ICV) x 100 (%).

The FDR correction was performed to verify the multiple comparisons for which a significance level with a *q* value of FDR correction was defined as <0.10.

The values were adjusted for age, sex, education, systolic blood pressure, use of antihypertensive agents, diabetes, serum total cholesterol, body mass index, cerebrovascular lesions on magnetic resonance imaging, smoking habits, drinking habits, regular exercise, and axial length.

## Discussion

The present study demonstrated that lower GC-IPL thickness estimated by using SS-OCT was significantly associated with greater likelihood of the presence of dementia in a general older Japanese population. In the imaging analysis using VBM, we also found that lower GC-IPL thickness was significantly associated with the brain volumes of brain regions related to cognitive function (i.e., the hippocampus, amygdala, entorhinal area, and parahippocampal gyrus) as well as visual function (i.e., the lingual gyrus, cuneus, and thalamus). Similar significant associations were also observed between GC-IPL thickness and the brain volumes of the hippocampus, amygdala, cuneus, lingual gyrus, and thalamus in the automated segmentation analysis using FreeSurfer software, with the beta coefficient of association for the hippocampus being the largest. Our findings suggested that GC-IPL thickness might reflect specific brain regional alterations related to cognitive function and subsequent cognitive dysfunction.

In the present study, lower GC-IPL thickness was significantly associated with a higher likelihood of the presence of all-cause dementia and AD, but RNFL thickness was not. No population-based study has assessed the association between GC-IPL or RNFL thickness by using SS-OCT and the presence of subtypes of dementia. Only 2 population-based studies have examined the association between inner retinal thickness as measured using spectral-domain OCT and all-cause dementia. The Rotterdam Study and the Japan Public Health Center Study revealed that the ORs for the presence of all-cause dementia significantly increased with lower GC-IPL thickness, whereas there was no evidence of a significant association between RNFL thickness and the presence of all-cause dementia in either study.^12^^,^^13^ Our findings were consistent with the results of these previous studies. However, a meta-analysis of clinical studies with AD patients reported that both GC-IPL and RNFL thickness measured using spectral-domain OCT were significantly associated with prevalent AD.^17^ This discrepancy in the findings was probably due to differences in the study design (population-based vs. case-control). With regard to the association between GC-IPL thickness and brain atrophy, only the Rotterdam Study reported a significant association between thinning of the GC-IPL and brain atrophy in the visual function areas (the cuneus, lingual gyrus, and thalamus).^24^ Meanwhile, the present study demonstrated that lower GC-IPL thickness was significantly associated with a lower volume of several brain regions related to not only visual function (the cuneus, lingual gyrus, and thalamus) but also cognitive function (the hippocampus, amygdala, entorhinal area, and parahippocampal gyrus). The discrepancy in findings between the Rotterdam Study and our present study may be due to the differences in the accuracy of the retinal thickness-measurement methods (SS-OCT vs. spectral-domain OCT) or of the study populations. In the present study, the significant positive associations between GC-IPL thickness and the volumes of brain regions related to cognitive and visual function were confirmed by using another tool for quantitative measurement of the brain volume, that is, the FreeSurfer software. Taken together, the present findings suggest that lower GC-IPL thickness may be associated with specific brain regions related to not only visual function but also cognitive function.

The exact mechanisms underlying the association of GC-IPL thickness with the likelihood of the presence of dementia and regional brain volumes are unclear. There are embryological, anatomic, and physiological similarities between the retina and brain.^46^^,^^47^ Animal studies using an AD model mouse have reported that depositions of amyloid-β and phosphorylated tau protein were found in the inner retina, especially in the retinal ganglion cell layers,^5^, ^6^, ^7^, ^8^, ^9^ and postmortem human brain studies also found these neurodegenerative changes in the inner retina.^10^^,^^11^ Notably, in the present study, the lower GC-IPL thickness was significantly associated with a lower volume of the hippocampus. A neuropathological study using AD model mice reported that the deposition of amyloid-β in the hippocampus appeared at almost the same time as that in the retina,^6^ suggesting that the progression of dementia may involve not only neurodegeneration of the brain, especially the hippocampus, but also neurodegeneration of ganglion cells of the retina. As another potential mechanism, there may be a link between retinal thickness and vascular damages reflected in white matter hyperintensities,^48^, ^49^, ^50^ because several epidemiological studies reported significant associations between a thinning of the retina and vascular risk factors such as diabetes and hypertension.^51^, ^52^, ^53^ However, in the present study, much as in the Rotterdam Study,^20^ there was no evidence of a significant association between each inner retinal thickness and white matter hyperintensities. Therefore, these findings raise the possibility that the thickness of retinal ganglion cell layers, such as the GC-IPL, may be associated with neurodegenerative changes rather than vascular damages in the brain.

### Study Limitations

The strengths of our study are the population-based design, the large sample size with advanced SS-OCT measurement, the use of 2 different analysis techniques for the evaluation of brain volume (i.e., VBM and FreeSurfer), the accurate determination of dementia by expert psychiatrists, and the detailed evaluation of confounding factors. However, there are potential limitations that should be noted. First, the current study was a cross-sectional study, and thus an interpretation of the causal relationship between GC-IPL thickness and prevalent dementia or brain atrophy could not be made. Second, there was a possibility of a selection bias caused by the exclusion of individuals from the present study. That is, excluded individuals were more likely to be of advanced age and more likely to have dementia than the included individuals (data not shown). This might have resulted in an underestimation of the observed associations. Third, information on hormonal imbalances (e.g., thyroid disease), vitamin deficiency, psychiatric illness, and substance abuse was not available in this study. Fourth, we were not able to conduct detailed analyses of the subtypes of dementia other than AD because of the limited number of non-AD events (n = 5). Fifth, the generalizability of the present findings may be limited, because this study was conducted in a local community of Japan. Therefore, the findings of this study should be validated in other regions and countries.

## Conclusions

The present data revealed that lower GC-IPL thickness was significantly associated with a higher likelihood of the presence of dementia and lower volumes of several brain regions related to cognitive or visual function. Our findings suggest that the measurement of GC-IPL thickness by an SS-OCT, which is a noninvasive, convenient, and reproducible device, might be a useful image marker for identifying high-risk individuals with dementia. Further prospective and experimental studies are required to validate the findings of this study, particularly the observed association between GC-IPL thickness and risk of dementia, and the potential use of GC-IPL thickness to predict the development of dementia.
