# Supplementary material for: Association of Inner Retinal Thickness with Prevalent Dementia and Brain Atrophy in a General Older Population: The Hisayama Study
Source: Ophthalmol Sci. 2022 Apr 19;2(2):100157. doi: 10.1016/j.xops.2022.100157 (PMC9559916; doi:10.1016/j.xops.2022.100157)
Supplement: Supplemental Table S1 [file mmc1.pdf]

Supplemental Table 1. The subtypes of dementia among 61 individuals of dementia, 2017

	n
Alzheimer's disease related cases (n=56)	
Alzheimer's disease	52
Mixed type of Alzheimer's disease and vascular dementia	3
Mixed type of Alzheimer's disease and dementia with Lewy bodies	1
Other subtypes of dementia related cases (n=5)	
Vascular dementia	2
Dementia with Lewy bodies	1
Hypoxic ischemic encephalopathy	1
Frontotemporal lobar degeneration	1
