# Supplementary material for: Association of Inner Retinal Thickness with Prevalent Dementia and Brain Atrophy in a General Older Population: The Hisayama Study
Source: Ophthalmol Sci. 2022 Apr 19;2(2):100157. doi: 10.1016/j.xops.2022.100157 (PMC9559916; doi:10.1016/j.xops.2022.100157)
Supplement: Supplemental Table S2 [file mmc2.pdf]

Supplemental Table 2. Odds ratio for the presence of all-cause dementia per every 1 standard deviation decrement in the GC-IPL and RNFL thickness in each of the right and left eyes for the entire eye and for the sectors related to the fovea and optic disc, 2017

	Right eye		Left eye	
	Multivariable-adjusted [a] OR (95% CI) per every 1 SD decrement	*p*	Multivariable-adjusted [a] OR (95% CI) per every 1 SD decrement	*p*
GC-IPL thickness				
Entire eye	1.62 (1.30 to 2.01)	< 0.001	1.63 (1.31 to 2.01)	< 0.001
Each sector				
Superotemporal	1.43 (1.15 to 1.79)	0.001	1.51 (1.23 to 1.85)	< 0.001
Superior	1.31 (1.03 to 1.66)	0.03	1.47 (1.17 to 1.85)	0.001
Superonasal	1.50 (1.21 to 1.87)	< 0.001	1.53 (1.23 to 1.91)	< 0.001
Inferonasal	1.55 (1.25 to 1.92)	< 0.001	1.45 (1.19 to 1.77)	< 0.001
Inferior	1.53 (1.23 to 1.91)	< 0.001	1.53 (1.23 to 1.90)	< 0.001
Inferotemporal	1.62 (1.31 to 2.01)	< 0.001	1.45 (1.19 to 1.77)	< 0.001
RNFL thickness				
Entire eye	1.26 (0.96 to 1.68)	0.10	1.19 (0.85 to 1.66)	0.31
Each sector				
Temporal	1.23 (0.90 to 1.69)	0.20	1.29 (0.88 to 1.91)	0.19
Superior	1.20 (0.92 to 1.57)	0.18	1.10 (0.82 to 1.48)	0.51
Nasal	1.12 (0.85 to 1.47)	0.43	0.74 (0.51 to 1.08)	0.12
Inferior	1.35 (0.96 to 1.89)	0.10	1.30 (0.91 to 1.87)	0.15

Abbreviations: GC-IPL, ganglion cell-inner plexiform layer; RNFL, retinal nerve fiber layer; SD, standard deviation; OR, odds ratio; CI, confidence interval.
a) The values were adjusted for age, sex, education, systolic blood pressure, use of antihypertensive agents, diabetes, serum total cholesterol, body mass index, cerebrovascular lesions on magnetic resonance imaging, smoking habits, drinking habits, regular exercise, and axial length.
